# Supplementary material for: Heterogeneous, delayed-onset killing by multiple-hitting T cells: Stochastic simulations to assess methods for analysis of imaging data
Source: PLoS Comput Biol. 2020 Jul 13;16(7):e1007972. doi: 10.1371/journal.pcbi.1007972 (PMC7386628; doi:10.1371/journal.pcbi.1007972)
Supplement: S1 Table — Data and code used in this project are available (http://doi.org/10.17605/OSF.IO/6GQYP). (PDF) [file pcbi.1007972.s001.pdf]

S1 Table

**Figures**	**Parameters**	**N_w (CTLs)**	**N_s (repeats per setting)**	**n (targets, t=0)**	**Model**
**1A,C**	$\eta = 1; 2;\ or\ 10,\ \eta = \lambda$	100	10	1	All-at-risk
**1D-E**<br>**2A***	$\eta = 1; 2;\ or\ 10,\ \eta = \lambda$	5000<br>10*	2 (12 or 24 hours).	$\sim Pois(16)$	All-at-risk
**2A**	$\lambda = 10,\ \eta = 10,$<br>$k_{on} = 1,\ k_{off} = 0.3$	10	1	$\sim Pois(16)$	Dynamic conjugate formation
**2C-F**<br>**S2**	(as $\eta,\ \lambda,\ k_{on},\ k_{off}$):<br>$(1,\ 0.34,\ 9,\ 0.75)$;<br>$(2,\ 1.21,\ 10,\ 1.00)$;<br>$(3,\ 2.15,\ 12,\ 0.00)$;<br>$(4,\ 3.04,\ 8,\ 0.25)$;<br>$(5,\ 3.93,\ 7,\ 0.50)$;<br>(10, 8.60, 3, 0.00).	10^3(fitting)<br><br>10^4 (figures / validation)	Until convergence (fitting)<br><br>1 (figures / validation)	$\sim Pois(16)$	Dynamic conjugate formation
**3-5**	$\eta = 1, 2,\ or\ 10, \eta = \lambda$<br>No hitting delay	100	1	$\sim Pois(16)$	Agent Based
**6, S6**	$\eta = 1, 2,\ or\ 10,\ \eta = \lambda$<br>Delay: 3-15min	100	1	$\sim Pois(16)$	Agent based,
**S1**	$\eta = 1,\ \lambda = 0.36$;<br>$\eta = 2,\ \lambda = 1.22$;<br>$\eta = 10,\ \lambda = 10$;<br>(base parameters)	1000	1	16	All-at-risk
**S3**	$\eta = 1,\ \lambda = 0.34$;<br>$\eta = 2,\ \lambda = 1.17$;<br>$\eta = 3,\ \lambda = 2.12$;<br>$\eta = 4,\ \lambda = 3.14$;<br>$\eta = 5,\ \lambda = 4.22$;	100	1	12	All-at-risk
**S4-5**	$\eta = \lambda = 10$;<br>$\eta = 1,\ \lambda_{LR} = 0.2,$<br>$\lambda_{HR} = 0.7$ (mixed population).	30 or 100	20 and 20	$\sim Pois(16)$	All-at-risk
