# Supplementary material for: Heterogeneous, delayed-onset killing by multiple-hitting T cells: Stochastic simulations to assess methods for analysis of imaging data
Source: PLoS Comput Biol. 2020 Jul 13;16(7):e1007972. doi: 10.1371/journal.pcbi.1007972 (PMC7386628; doi:10.1371/journal.pcbi.1007972)
Supplement: S1 Text — (PDF) [file pcbi.1007972.s002.pdf]

Supplementary Text

Fitting procedure for cumulative interaction times

In all our models the underlying dynamics of the CTL-target killing process remain constant over time. CTLs hit targets at a characteristic rate λ, with targets dying after a set number of hits η. The models differ in the dynamics of CTLs contacting targets, as well as the rules governing which contacted targets are hit. Our aim was to devise a method of recovering the underlying killing-associated parameters of our models (i.e., λ and η), regardless of which model they originated from, which could in future be used to analyse *in vitro* or *in vivo* data. In each simulation we have N_w independent simulated wells each containing n target cells and a single CTL. Thus, the total number of targets n_{tot} equals:

$$n_{tot} = \sum_{i=1}^{N_w} n_i \ , \qquad \text{Eq S1}$$

with the index $i=1,2,...N_w$ reserved for individual CTLs. Simulations are sampled at intervals Δt, typically ranging from 1 to 15 minutes. Rather than examining each of the wells separately, in our analysis we treat each of the n_{tot} targets as an individual data point. Thus, application of our approach requires accurate tracking of individual targets. In our simulations the probability of a target cell being hit at any moment is $\lambda\theta \cdot dt$. Here, the dimensionless quantity θ represents the probability that any individual target is being attacked by the CTL and is independent of the hitting rate λ. In other words, θ represents the momentary probability that a CTL is attacking a specified individual among c simultaneously contacted targets, so we consider:

$$\sum_{i=1}^{c} \theta_{it} = 1 \ , \qquad \text{Eq S2}$$

i.e. the probability of hits delivered from one CTL to all c contacted targets is 1, with θ_{it} the probability of hitting an individual target i. The t indexes the sample times $t = \{t_1, t_2, t_3, ...T\}$, where T is the duration of the simulation. Note that we consider $\theta = 0$ for targets either dead or not in contact with a CTL. In general, CTLs may attack contacted targets with uneven probability. However, because our aim was to recover accurate estimates for λ and η from a general set of interactions, we approximate θ_{it} individually for every simulated target by considering that all contacted targets are equally at risk of being hit. Thus:

$$\theta_{it} = \frac{1}{c_{it}} , \qquad \text{Eq S3}$$

where c_{it} is the total number of targets co-contacting the CTL at the moment the sample is taken. Therefore our analysis treats c_{it} as the same for all targets co-contacting one CTL at any moment. Note that in our CPM simulations CTLs do not distribute hits with equal probability to all targets; instead the instantaneous probability of a specific target receiving a hit depends on the physical size of that target's interaction interface with the CTL. Moreover, in some cases we restrict targets from being hit for a (variable) delay period whenever they contact (or recontact) a CTL. Thus, with the CPM simulations we can verify whether the fitting procedure is robust to settings in which CTL attention is not distributed equally amongst contacted targets. We define the quantity τ as the cumulative time an individual target has spent in-contact with a CTL (scaled according to Eq S3) during a simulation lasting for T hours, so for an individual target:

$$\tau = \int_0^T \theta \, dt , \qquad \text{Eq S4}$$

i.e. τ is a continuous variable with units of time representing the amount of time a CTL interacted with a target, divided by the number of other co-contacting targets throughout time.

The interaction history of each target cell provides us with one sample of τ and for time lapse imaging data this needs to be estimated from discrete observations with regular interval Δt:

$$x_{ij} = \sum_{t=t_1}^{T} \theta_{ijt} \cdot \Delta t \ , \qquad \text{Eq S5}$$

where x_{ij} is one sample and the index $j_i = 1,2,...n_i$ is used for individual targets belonging to the ith CTL. We refer to the set of all samples of τ from a single simulation as X, so that $x_{ij} \in X$. For our analysis we consider the subset of target cells which survived until the end of the simulation $S \subseteq X$ (containing samples s_{ij}) separately to those samples where target cells were killed, $K \subseteq X$ (containing samples k_{ij}). To make inferences about the CTL killing dynamics, we would ideally like to use both the samples of surviving (S) and killed targets (K), thus taking into account all the information available. We define a maximum likelihood estimate for the model parameters that maximises the joint probability of all of the samples $P_{K \cap S}$:

$$P_{K \cap S} = \prod_{k_{ij} \in K} P_K(k_{ij}) \prod_{s_{ij} \in S} P_S(s_{ij}) \ , \qquad \text{Eq S6}$$

where $P_K(k_{ij})$ is the probability of observing any killed sample $k_{ij} \in K$ individually, and $P_S(s_{ij})$ is the probability of observing any surviving sample $s_{ij} \in S$ individually. The remaining task is to find functions expressing P_K and P_S in terms of the parameters for the CTL hitting process, i.e. η and λ. If CTLs are given sufficient time to kill all targets, the waiting times until death for all the samples forming the set of killed targets K are distributed according to the gamma probability density function $f_k(\tau|\eta,\lambda)$, with a shape parameter η and a rate parameter λ (S3A Fig, red line). When some targets survive however, the distribution of the killed targets k_i will change (S3B Fig), since any surviving targets are absent from it (S3C Fig). For certain ideal cases with simple rules, exact equations defining the distributions for all targets have been

derived in terms of Markov chains [1, 2]. However for larger numbers of targets and more complex scenarios, analytical solutions become intractable. Instead our aim here was to develop a more general approach that is applicable to a broad range of experimental settings.

Consider the location one would expect a surviving target (with total attention time s_{ij}) to fall in the distribution of killed targets, had the simulations indeed continued until it was killed. According to our probability function $f_k(\tau|\eta,\lambda)$, this hypothetical killing time should be "spread" over all τ values greater than s_{ij}. This spreading should be done with a weight w_{ij} following $f_k(\tau|\eta,\lambda)$, defined as:

$$\int_{s_{ij}}^{\tau} f_k(\tau|\eta,\lambda)\cdot d\tau = \frac{1}{w_{ij}}, \qquad \text{Eq S7}$$

so that observed s_{ij} are giving equal weighting to k_{ij}, i.e. unity. The integral of $f_k(\tau|\eta,\lambda)$ is the gamma cumulative distribution function, $F_k(\tau|\eta,\lambda)$. Because Eq. S7 can be solved explicitly: $\int_{s_{ij}}^{\tau=\beta} f_k(\tau|\eta,\lambda)\cdot d\tau = F_k(\beta|\eta,\lambda) - F_k\left(s_{ij}|\eta,\lambda\right)$, an expression for the value of each weighting term w_{ij} can be written as follows:

$$w_{ij} = \frac{1}{F_k(\beta|\eta,\lambda) - F_k\left(s_{ij}|\eta,\lambda\right)}. \qquad \text{Eq S8}$$

Because β is constant and $\beta > s_{ij}$ for a specified η,λ, we may assume there exists a weighting function $\varphi_{\eta,\lambda}(\tau)$, with:

$$\varphi_{\eta,\lambda}(\tau)\cdot\left(1 - F_k\left(s_{ij}|\eta,\lambda\right)\right) = \frac{1}{w(s_{ij})}, \qquad \text{Eq S9}$$

which importantly allows the samples to be broken into two components: one expressed in terms of the samples, $w(s_{ij})$, and the second, $\varphi_{\eta,\lambda}(\tau)$, a correcting function for a specific set of η and λ. With a functional expression of these weightings in hand we can proceed to write the hypothesized probability density function, $f_k(\tau|\eta,\lambda)$, in terms of the modified distribution of CTL attention times for killed cells, $P_K(\tau|\eta,\lambda)$, plus our gamma distributed forecast based on the surviving cells s_{ij}:

$$f_k(\tau|\eta,\lambda) = P_K(k_{ij}|\eta,\lambda) + \varphi_{\eta,\lambda}(\tau)\left(1 - F_k\left(s_{ij}|\eta,\lambda\right)\right). \qquad \text{Eq S10}$$

Here, $\varphi_{\eta,\lambda}(\tau)$ is a function containing information about other factors influencing the distribution of adjusted cumulative contact times (τ) for killed or surviving cells, for example CTL motility, if such knowledge is present and one wishes to include it in the model. For our analysis we are seeking a general solution for recovering the hitting parameters λ and η, so we treat $\varphi_{\eta,\lambda}(\tau)$ as a nuisance variable that we aim to remove from the analysis without damaging our fitting results. Note that if we hold $\varphi_{\eta,\lambda}(\tau)$ constant, we have a function containing all the samples depending only on our parameters. Although by discarding any ancillary information contained in $\varphi_{\eta,\lambda}(\tau)$ we do not estimate the absolute probability of a set of observations, by holding $\varphi_{\eta,\lambda}(\tau)$ constant we may still accurately determine the parameters λ and η. That is because, having isolated the components of our model that depend on the unknown parameters, we have obtained a sufficient parametric division of our problem (see also: [3], appendix 3). In practise this means that we can hold the residual likelihood φ as a constant during fitting, thus allowing the probability of our samples to be expressed:

$$P_{K\cap S} = \psi \prod_{k_i \in K} f_k(k_i|\eta,\lambda) \prod_{s_i \in S} \left(1 - F_{k,\eta,\lambda}(s_i)\right), \qquad \text{Eq S11}$$

with ψ indicating that due to the discarded information $\varphi_{\eta,\lambda}(\tau)$ we have not computed the absolute probability $P_{K\cap S}$, but only found the parameters η and λ which maximise $P_{K\cap S}$. Note that this works because during the estimation process the samples k_i and s_i are fixed whilst η and λ are varied, therefore the contribution of the residual error remains the same. However, the success of the parameter estimation overall does depend on the extent to which the summary statistic τ depends on the information discarded.

Fitting procedure for subpopulations of single-hitting CTLs

It is desirable to have a procedure to compare the likelihood of multiple-hitting to other hypotheses. An alternative to the multiple-hitting hypothesis, which may also describe the emergence of high-rate killing over time, is the subpopulation model originally suggested by Vasconcelos *et. al.* [4]. In the subpopulation model high-rate killing is due to a CTL subpopulation that has an intrinsic capability to kill at high rates. In order to compare the subpopulation with the multiple-hitting hypothesis, we followed the strategy of Vasconcelos *et. al.* [4], who sampled the final number of killed targets per-CTL after the 12 hour experiments, then fit a Poisson mixture model to the resultant distribution. However, different to their fitting approach, we utilised a maximum likelihood approach which also integrated the initial number of targets. This takes explicit account of possible censorship of the data (e.g. due to CTLs exhausting the supply of targets). Furthermore, the initial number of targets together with the number of kills per CTL contains information about the number of surviving targets per well, making comparisons with the multiple-hitting model more straightforward.

Our first step was to recover the hitting rate parameter λ for uniform populations of single-hitting CTLs ($\eta = 1$). For such a uniform CTL population, λ can be inferred by maximising the

likelihood of each number of targets killed per CTL according to an n-truncated Poisson model. If a CTL had available *n* targets, then the probability of observing *x* kills is:

$$P\ (x;\ x < n) = pois(x,\ \lambda)\ , \qquad \text{Eq S12}$$

if $x < n$. When $n = x$, the probability is given by:

$$P\ (x = n) = 1 - \int_{0}^{n-1} pois(x,\ \lambda)\ , \qquad \text{Eq S13}$$

which is easily determined since the integral is the Poisson cumulative distribution function. Note that Eq S13 (for $n = x$) is a natural consequence of our basic requirement that the probability distribution should integrate to unity. To extend the Poisson fitting method to a situation with a subpopulation of high-rate killers amongst low-rate killers, we maximise the likelihood of the samples according to a 2-component, weighted, n-truncated Poisson mixture. The probability of a single sample can be expressed for the ith CTL:

$$P\ (x_i; m, \lambda_{HR}, \lambda_{LR}) = m \cdot P\ (x_i, \lambda_{HR}) + (1 - m) \cdot P\ (x_i, \lambda_{LR})\ , \qquad \text{Eq S14}$$

where P is the probability density function described in Eq S12-13, parameterised by the relevant rate parameter for either the high- or low-rate killing subpopulation. In the absence of mechanistic insight into the origins of the hypothesised subpopulations, we use a simple binomial model such that each CTL has a fixed prior probability of belonging to the high-rate killing population. This probability is referred to as the mixture parameter *m*, a constant to be estimated. The maximum likelihood estimates ($\widehat{m}, \widehat{\lambda}_{HR}$, $\widehat{\lambda}_{LR}$) are the values taken by the parameters which maximise the joint probability of a given set of samples (*x)*:

$$\widehat{m},\ \widehat{\lambda}_{HR},\ \widehat{\lambda}_{LR} \mid x = \ argmax \prod_{i=1}^{N_w} P_i(x; m, \lambda_{HR}, \lambda_{LR})\ . \qquad \text{Eq S15}$$

The estimation proceeds with randomly selected parameter estimates, which are then iteratively updated using an expectation maximisation procedure. Specifically, estimates for λ_{LR} and λ_{HR} were used to produce a posterior estimate for the weight of the ith sample w_i, which is the ratio:

$$w_i(x;\lambda_{HR},\lambda_{LR}) \sim \frac{P_i(x;\lambda_{HR})}{P(x;\lambda_{HR})+P(x;\lambda_{LR})}\ , \qquad \text{Eq S16}$$

The mean of the weights derived from λ_{LR} and λ_{HR} is an estimate m_{est} of the mixture parameter:

$$\frac{1}{N_w}\sum_{i=1}^{N_w} w_i(x_i;\lambda_{HR},\lambda_{LR}) = m_{est} \sim B(m)\ , \qquad \text{Eq S17}$$

with m_{est} treated as a realisation of a random variable drawn from the population whose true fraction (of CTLs with $\lambda=\lambda_{HR}$) is the parameter m. This has the binomial probability $B(m)$ which can be used to constrain the parameter m in the fit. Our maximum likelihood estimates for the mixture parameters maximised the log likelihood function:

$$\widehat{\lambda}_{HR},\widehat{\lambda}_{LR},\widehat{m}\mid x = \ argmax\ N_w\ log\ B(m=m_{est};\ \lambda_{HR},\lambda_{LR}) + log\ \sum_{i=1}^{N_w} w_{\lambda_{LR},\lambda_{HR}}\ P\ (x_i;\lambda_{HR}) + (1-w_{\lambda_{LR},\lambda_{HR}}) + P\ (x_i;\lambda_{LR})\ . \qquad \text{Eq S18}$$

Comparing Multiple-hitting vs subpopulation hypothesis

To compare hypotheses we used the Monte-Carlo simulations where all targets shared risk equally throughout the simulations (as in main Text Fig. 1). We retained the previously tested parameters $\eta = 10$, λ=10 (hr^{-1}) to represent the multiple-hitting hypothesis. In addition, we simulated two groups of single-hitting CTLs $(\eta = 1)$ to represent the subpopulation hypothesis. One group of single-hitting CTLs had a relatively low killing rate λ_{LR}= 0.2(hr^{-1}), the second group a higher killing rate λ_{HR}=0.7(hr^{-1}). For each group we performed 2 rounds of N_w=1000 simulations, each round starting with a different mean $\bar{n}$ for the Poisson-distributed initial number of targets per-CTL. One round of simulations started with mean $\bar{n} = 8$ (S4A Fig, left column), and the other with $\bar{n} = 16$ (S4A Fig, right column).

The $\bar{n} = 8$ condition resulted in heavy censorship of the data for multiple-hitting CTLs and for single-hitting CTLs with λ_{HR}=0.7(hr^{-1}), because in both cases the majority of CTLs killed all of their targets (S4B Fig, left column). We simulated the $\bar{n} = 8$ condition to ensure that our procedure would handle such censored samples well and these $\bar{n} = 8$ simulations were not used for testing the mixed population. Subsequently, we combined the groups with $\eta = 1$ in order to generate a mixed dataset representing the subpopulation hypothesis (S4C Fig). As before, the mixing parameter *m* represents the fraction of the mixed population with λ_{LR}= 0.2(hr^{-1}), such that the remainder *(1-m)* belongs to the λ_{HR}=0.7(hr^{-1}) group. The mixing parameter *m* was selected for the $\bar{n} = 16$ conditions such that the mean killing from the resultant mixed dataset would be the same as the mean killing for the multiple-hitting CTLs, which was achieved with *m=0.67* for simulations with $\bar{n} = 16$. The resulting mixed distributions representing the subpopulation hypothesis (S4C Fig), are similar to the multiple-hitting $(\eta = 10)$ groups (compare to S4B Fig, bottom right).

We first established that our Poisson estimated rate parameters (denoted $\widehat{\lambda}$) would be accurate for the individual rate parameters $\boldsymbol{\lambda}_{LR}$ and $\boldsymbol{\lambda}_{HR}$ when fitted separately (S4D Fig). As expected, for large sample numbers (N_w=1000) the Poisson estimators were reliable (S4D Fig, dashed lines). We also tested a smaller sample number (N_w=10; S4D Fig, solid lines), for which the bounds on the estimated $\widehat{\lambda}$ values were much less constrained, particularly for the $\boldsymbol{\lambda}_{HR}$=*0.7* group compared to the $\boldsymbol{\lambda}_{LR}$=*0.2* group (S4D Fig, right versus left column) and for the group with the lower $\bar{n}=8$ compared to the group with the higher $\bar{n}=16$ (S4D Fig, top versus bottom row). For N_w=10, the differences between simulations with different $\bar{n}$ and $\boldsymbol{\lambda}$ were due to the CTLs with rate $\boldsymbol{\lambda}_{HR}$ often eliminating all available targets, especially in those simulations with the low initial number of targets $\bar{n}=8$.

Having established that our Poisson estimator would perform well for large sample numbers even if the CTLs in some simulations eliminated all their targets, we did not consider the data for $\bar{n}=8$ further. Instead we applied both our estimator for the subpopulation model, as well as our estimator for the multiple-hitting model, to data generated by multiple-hitting CTLs (S4E Fig, right column), or data generated by mixed populations of CTLs with $\bar{n}$*=16* (S4E Fig, left column). For these tests, we used smaller “testing populations” assembled by drawing random members from the 3 larger populations shown (S4B Fig). Each testing population contained either solely multiple-hitting CTLs, or a mixture of single-hitting CTLs using the previously obtained mixture fraction *m=0.67.* Moreover, we tested differently sized populations with either N_w=100 (S4E Fig, bottom row), or N_W=30 CTLs per test (S4E Fig, top row). To begin the parameter estimation we first used constrained versions of the respective models. The constraints were set such that each model became the uniform population of single-hitting CTLs, which is nested within both models when (for the subpopulation model) $\lambda_{HR}=\lambda_{LR}=\lambda$ or

(for the multiple hitting model) $\eta = 1$ (S4F Fig). Because we took into account the surviving targets with the Poisson estimator, it asymptotically approached the Gamma estimator on the maximum likelihood estimate, $\widehat{\lambda}_{ML}$, for sufficiently large N_w (S4F Fig, bottom row). The difference between the Poisson estimators and the Gamma estimators is that the per-target samples, $x_{ij,}$ were transformed to per-CTL samples x_i for fitting the subpopulation model, thus losing any information pertaining to the killing rate of CTLs over time.

Next we estimated the parameters for all datasets using unconstrained versions of the Gamma estimator, which is looking for evidence of multiple-hitting, or the Poisson estimator, which seeks subpopulations (S5A Fig). The unconstrained Gamma estimator did not increase the likelihood of any of the subpopulation-generated testing data (S5B Fig, absence of dark bars), since it did not predict multiple-hitting unless multiple-hitting was present in the data (S5C Fig; note that only fits with $\eta > 1$ are annotated). However, when the unconstrained Gamma estimator was applied to the multiple-hitting-generated testing data, substantial improvements in likelihood resulted (S5B Fig, dark bars). Moreover, satisfactory estimates for the parameters were achieved (S5C Fig, annotated points). To better understand this result, we examined the cumulative interactions, τ, for all the samples in each generating dataset (S5D Fig). The multiple-hitting-generated data was spread over a much smaller range of τ than was the subpopulation-generated data. This reflects the fact that under multiple-hitting, very short interactions are unlikely to lead to enough hits for killing. This explains the reduced sample density of killed targets in the multiple-hitting-generated data in comparison to subpopulation-generated data, for the lowest values of τ (S5D Fig, "killed"). Very lengthy interactions are also unlikely for multiple-hitting CTLs, since targets that have been contacting a CTL for a long time become increasingly likely to have received hits. This explains the reduced

sample density of both killed and surviving targets in our datasets, comparing again multiple-hitting-generated to subpopulation-generated data, for the highest values of τ (S5D Fig, both columns). The subpopulation-generated data is in fact spread further over τ than would be the case for a uniform population, as the mixture of exponentials results in a hyperexponential distribution. Thus, the Gamma estimator prefers $\eta = 1$ strongly if there is a subpopulation of single-hitting CTLs, since $\eta > 1$ has an opposing effect i.e. it concentrates the distribution around the mean.

Considering now the unconstrained Poisson estimator; when applied to the subpopulation-derived data this also recovered satisfactory estimates for the different killing rates, λ_{LR} and λ_{HR} (S5C Fig, bottom row "subpopulation"). When the Poisson estimator was applied to the multiple-hitting-generated data, this produced similar estimates for $\widehat{\lambda}_{LR}$ and $\widehat{\lambda}_{HR}$ as for the subpopulation-generated data (S5B Fig, "subpopulation"), which was due to the similar distributions (S4B Fig). Thus, in this example there was a 100% false-positive rate.

Supplementary References

1. Macken CA, Perelson AS (1985) Some stochastic models in immunology. Acta Appl Math 4:157–200
2. Perelson AS, Macken+ A (1984) Kinetics of Cell-Mediated Cytotoxicity: Stochastic and Deterministic Multistage Models. Math Biosci 70:161–194
3. Therneau TM, Grambsch PM, Fleming TR (1990) Martingale-Based Residuals for Survival Models. Biometrika 77:147–160
4. Vasconcelos Z, Müller S, Guipouy D, Yu W, Christophe C, Gadat S, Valitutti S, Dupré L (2015) Individual Human Cytotoxic T Lymphocytes Exhibit Intraclonal Heterogeneity during

Sustained Killing. Cell Rep 11:1474–1485
