# Supplementary figures and images for: Heterogeneous, delayed-onset killing by multiple-hitting T cells: Stochastic simulations to assess methods for analysis of imaging data
Source: PLoS Comput Biol. 2020 Jul 13;16(7):e1007972. doi: 10.1371/journal.pcbi.1007972 (PMC7386628; doi:10.1371/journal.pcbi.1007972)

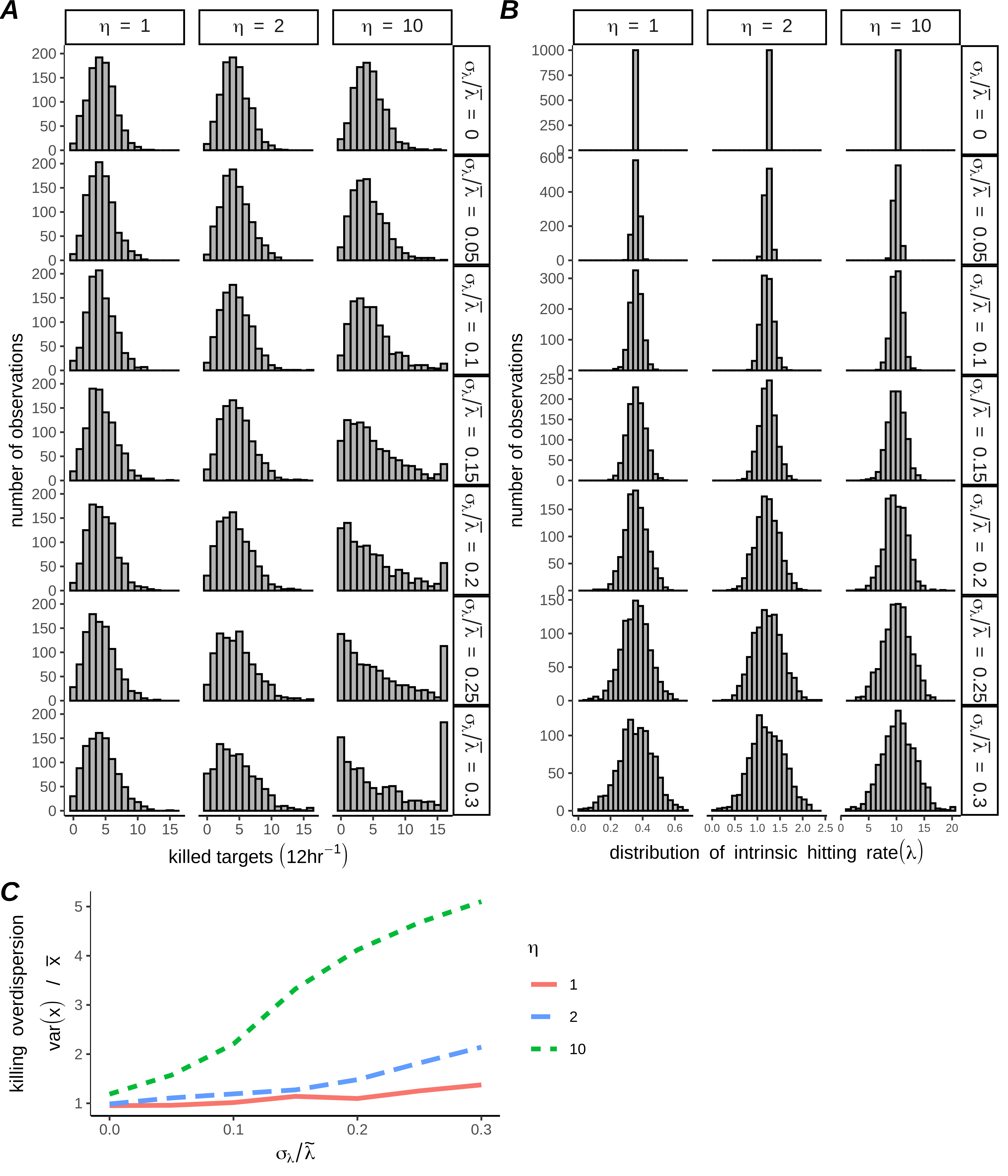

Supplement: S1 Fig — A-B) Distribution of killed target numbers after 12 hours (A) when intrinsic hitting rates λ (B) are drawn from a normal distribution with mean λ¯ and standard deviation σλ(λ∼Normal(λ¯,σλ)). C) Overdispersion for the variance in killed targets in A relative to the variance expected for a Poisson distribution, i.e., the ratio of the variance (var(x)) to the mean (x¯) number of targets killed after 12 hours (vertical axis). The horizontal axis is the ratio of the standard deviation to the mean value of the intrinsic hitting rate. (TIFF) [file pcbi.1007972.s003.tiff]

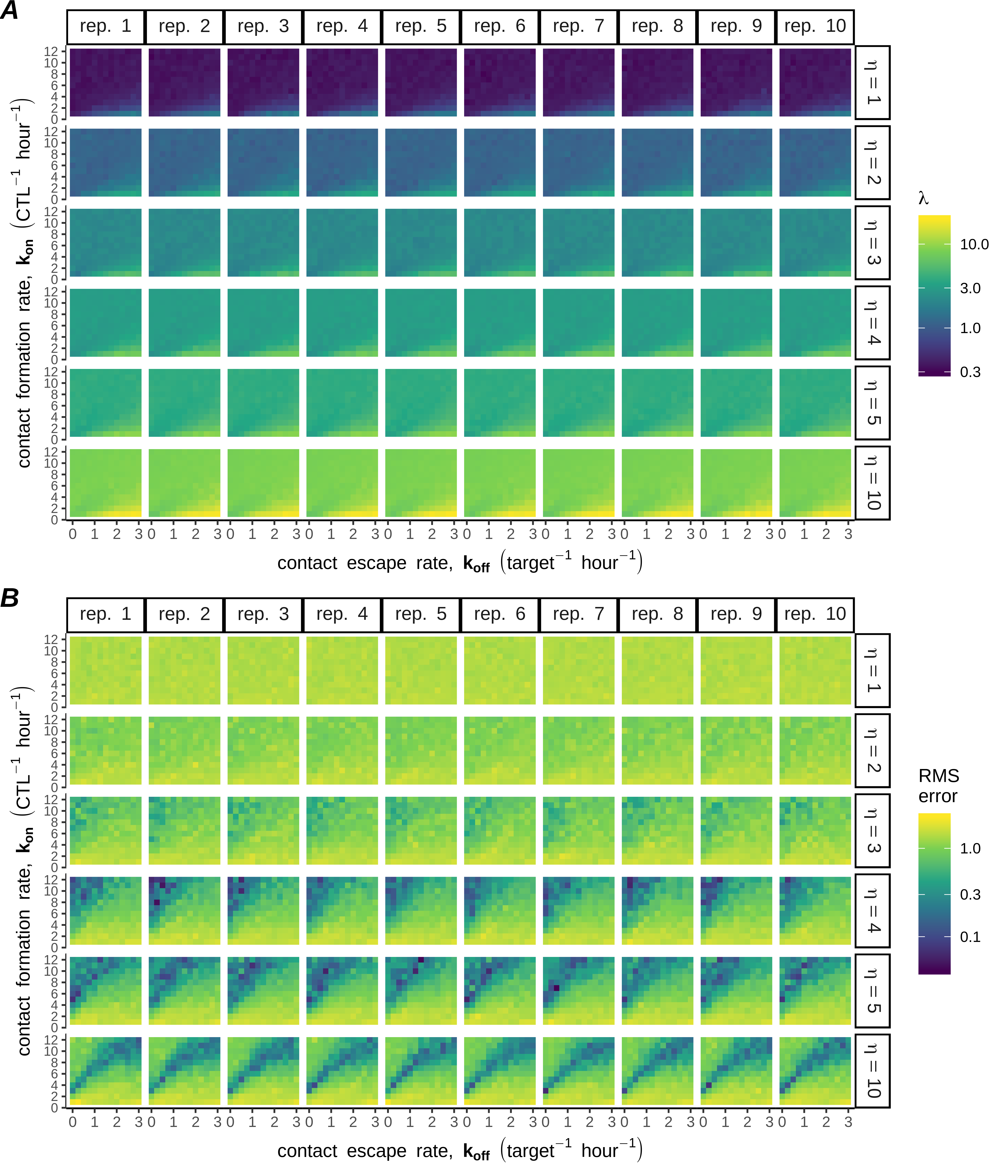

Supplement: S2 Fig — A) Estimated hitting rates (λ, represented by colour) for various combinations of the number of hits (η, rows), contact formation rates (kon, vertical axes in sub-panels), or contact escape rates (koff, horizontal axes in sub-panels). Ten repeats (across columns) were performed for the optimisation step, using Nw = 103 CTLs per tested value of λ. After fitting we validated our results by performing Nw = 104 simulations with each best fitting parameter combination, which is shown here. B) Root mean square residual errors for the best fitting parameter estimates (panel arrangement is as described in S2A Fig legend). Results are from validation simulations, using Nw = 104 simulations per parameter combination. (TIFF) [file pcbi.1007972.s004.tiff]

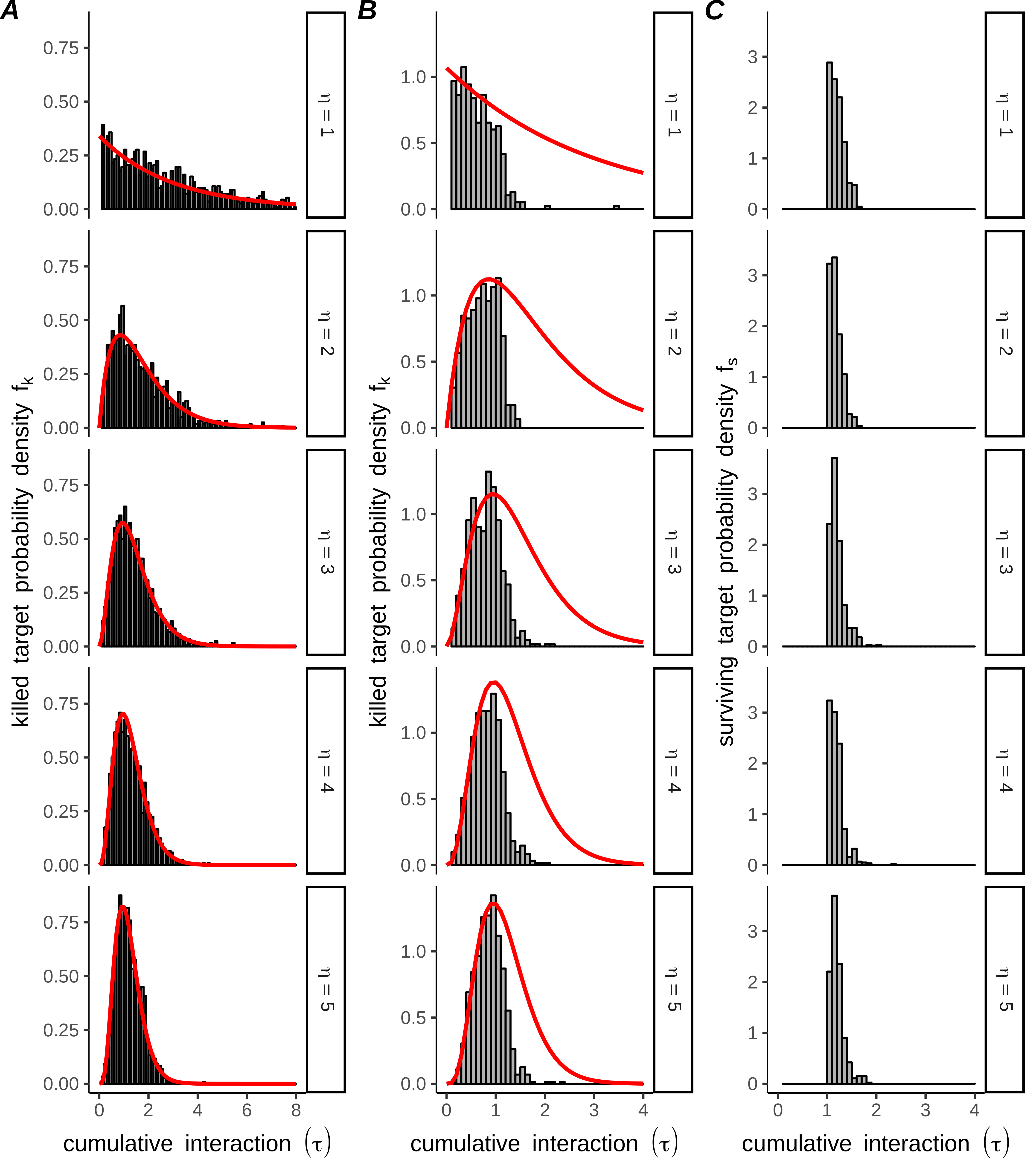

Supplement: S3 Fig — A) Sample density of killed targets in Monte Carlo simulations lasting until all targets were killed, with different numbers of hits (η, on different rows). B) Sample density of killed targets in Monte Carlo simulations stopped after 12 hours. C) Sample density of surviving targets, corresponding to the ‘absent’ portion of the distribution for killed targets in B. The red line in A and B is the function fk, which describes how the relative probability until targets receive η hits arriving at a constant rate λ depends on the cumulative interaction time τ, for a gamma distributed waiting time. For all S3 Fig: Nw = 100, n = 12 targets per well, all targets equally at risk. Parameter combinations used were: (η = 1,λ = 0.34; η = 2,λ = 1.17; η = 3,λ = 2.12; η = 4,λ = 3.14; η = 5,λ = 4.22). (TIFF) [file pcbi.1007972.s005.tiff]

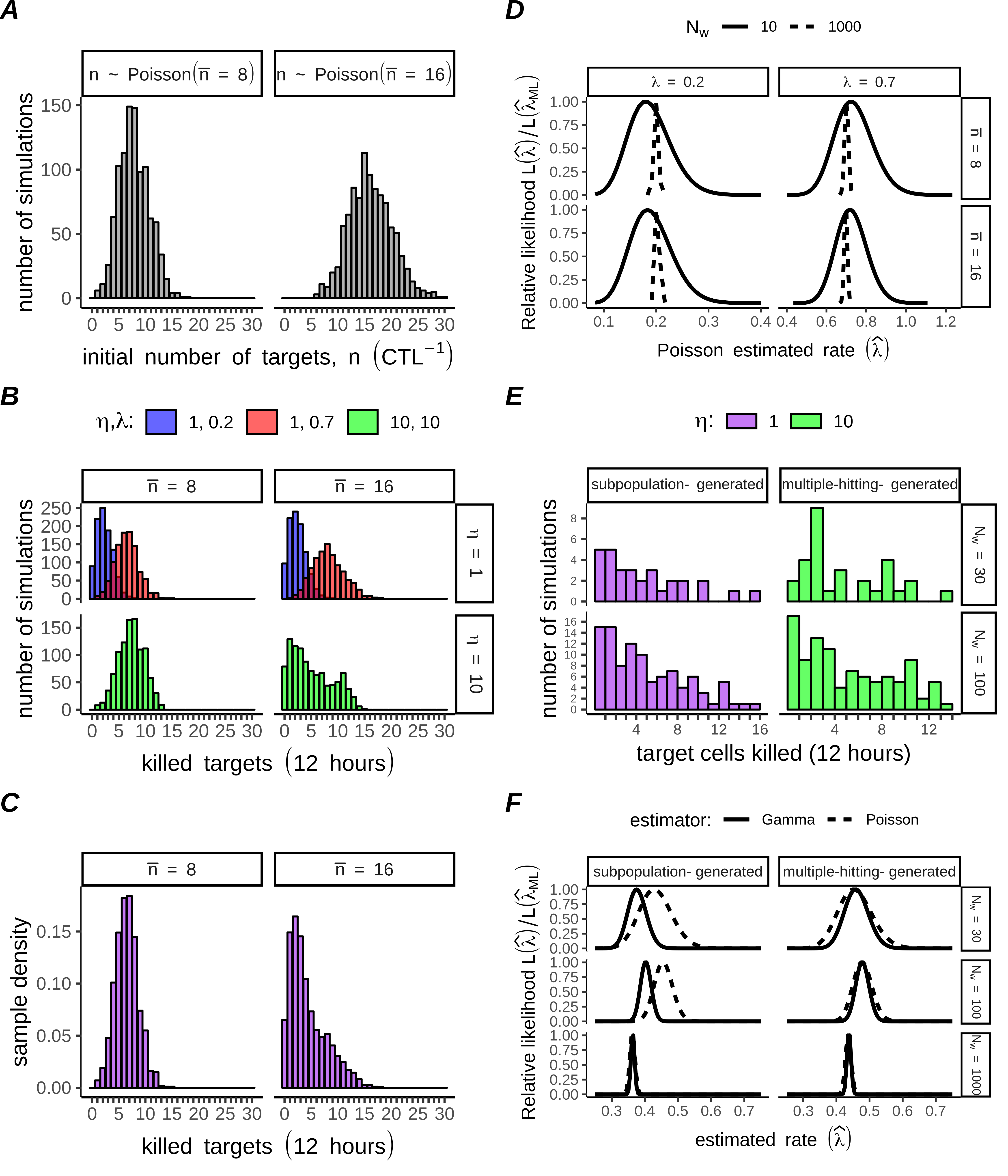

Supplement: S4 Fig — A) Poisson distributions for the number of targets used to start simulations in S4 Fig, with mean n¯=8or16 as shown. B) Number of killed targets after 12 hours for Nw = 2 x 3 x 1000 simulations, each group of Nw = 1000 started with one of the 2 distributions in A, and with one of the 3 indicated parameter settings. C) Density of killed targets after 12 hours from ‘Mixed’ distributions resulting from η = 1,λLR = 0.2,λHR = 0.7 and either n¯ = 8 and m = 0 (left panel), or n¯ = 16 and m = 0.67 (right panel). Note that for n¯ = 8 the killing of multiple-hitting CTLs became greater than the high rate subpopulation of single-hitting CTLs; n¯ = 8 was only used for testing robustness of the estimators on heavily censored data. D) Relative likelihood of candidate hitting rate estimates, λ^, compared to the maximum likelihood estimate, λ^ML, resulting from application of the Poisson estimator separately to each of the single-hitting (η = 1) datasets shown in B. Relative likelihood are shown either for the dataset in its entirety (dashed lines), or for a randomly selected sample of NW = 10 (solid lines). E) Examples of testing datasets derived from the multiple-hitting population (B, n¯ = 16, η = 10) or from a mixture of single-hitting CTLs (B, n¯ = 16, η = 1, where the true density of killed targets in the mixture distribution is in C). F) Relative likelihood of candidate hitting rate estimates, λ^, compared to the maximum likelihood estimate, λ^ML, for constrained fits constructed from either the subpopulation datasets, or from multiple-hitting datasets, for three samples with either Nw = 30,100, or 1000 (note the multiple-hitting-generated data (η = 10) is therefore fully represented by the Nw = 1000 case). (TIFF) [file pcbi.1007972.s006.tiff]

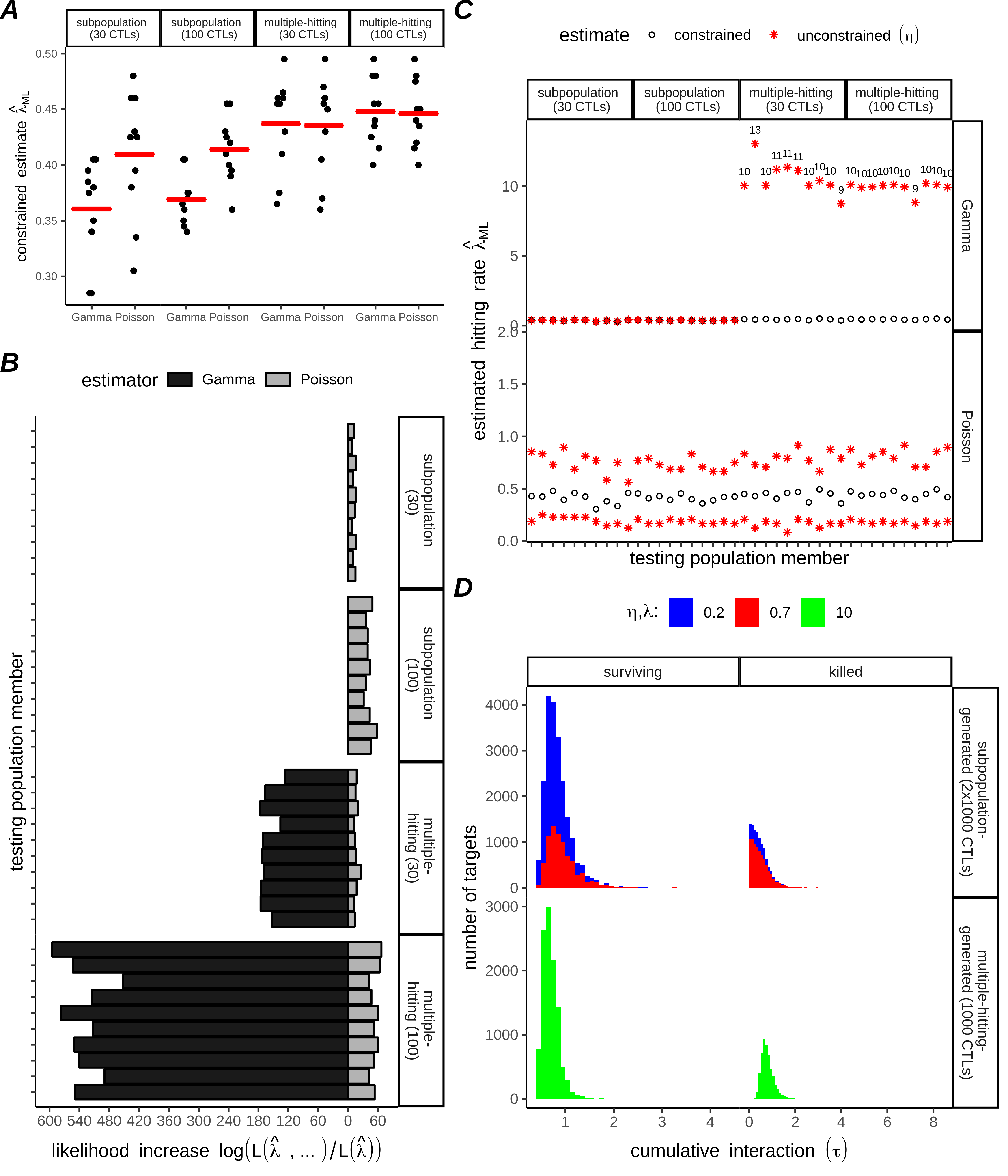

Supplement: S5 Fig — A) Maximum likelihood estimates for the hitting rate, λ^ML, with either the gamma or Poisson estimators, both constrained to a uniform single-hitting population (i.e. by forcing η = 1 for the gamma estimator and by forcing m = 1 for the Poisson estimator). Each of the 2x4x10 = 80 points represents one of the 4x10 testing populations from S4E Fig (here indicated by facet labels), fit with both of our estimators (x-axis). B) Difference between the log likelihood function evaluated with the constrained versus unconstrained Gamma estimator logL(λ^ML,η^ML) (dark bars); or with the constrained versus unconstrained Poisson estimator logL(λ^HR,ML,λ^LR,ML,m^ML) (light bars). Each of the 40 testing populations occupies one horizontal bar, with the details of the testing populations as indicated in facet labels. For the x-axis scaling (negative values are not possible), the relative size of the dark v.s. light bars is proportional to the strength of the evidence for the multiple-hitting hypothesis (dark bars) versus the subpopulation hypothesis (light bars). C) The constrained estimates for the hitting rate parameters, λ^ML, (circles; also shown in A) or their unconstrained counterparts (red asterisks) for each testing population (points on x-axis). For the Gamma estimator (top row) the estimated η^ML is shown only where η^ML>1. For the Poisson estimator (bottom row), the unconstrained estimates for λ^HR,ML,λ^LR,ML are above and below their counterpart constrained estimates, and the Gamma and Poisson estimators can be compared per population. D) Distribution of all cumulative interaction times, τ (killed and surviving targets shown separately in columns), for all Nw = 1000 members of each of the 3 generating populations (as shown in S4B Fig). Although the 2 single-hitting populations were combined (upper row), the separate contribution of the λHR (red) or λLR (blue) populations is indicated by color. Multiple-hitting CTLs (green) are shown separately (bottom row). (TIFF) [file pcbi.1007972.s007.tiff]

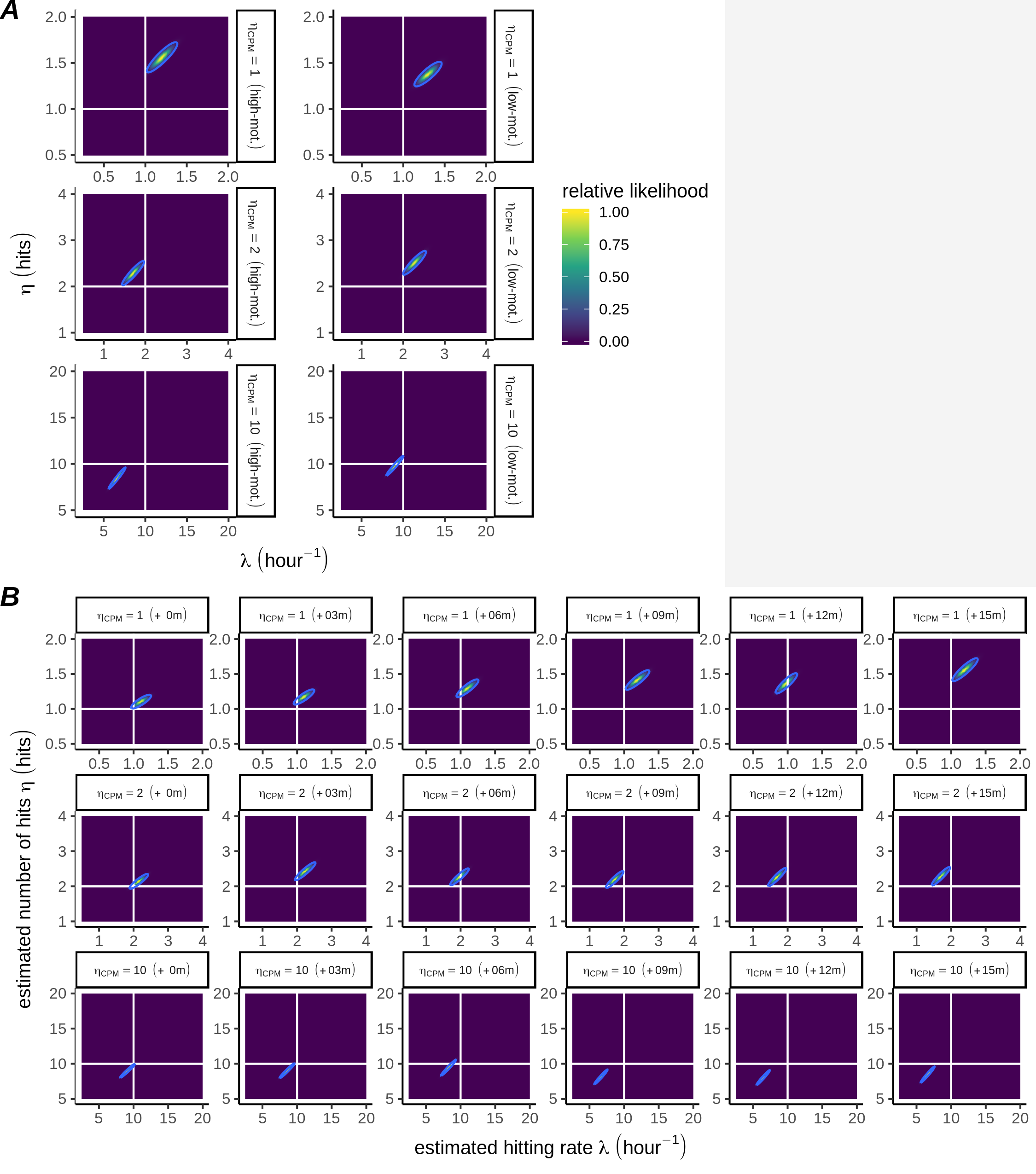

Supplement: S6 Fig — A-B) Heatmaps of the likelihood function around the maximum likelihood estimates for the killing parameters η and λ, in CPM simulations under various conditions. In A, results are shown for various η values (rows) and for both high-motility (left colum) and low-motility (right column) conditions for simulations with 15 minute hitting delay. In B, results are shown for high motility CTLs at all tested values of the delay (in range 0–15 minutes, across columns). The horizontal and vertical lines in A-B mark the values of the CPM parameters used to generate the data for each group and the boundary enclosing the 95% confidence region is marked with a thin blue line. (TIFF) [file pcbi.1007972.s008.tiff]
