# Supplementary material for: Heterogeneous, delayed-onset killing by multiple-hitting T cells: Stochastic simulations to assess methods for analysis of imaging data
Source: PLoS Comput Biol. 2020 Jul 13;16(7):e1007972. doi: 10.1371/journal.pcbi.1007972 (PMC7386628; doi:10.1371/journal.pcbi.1007972)
Supplement: Attachment — Submitted filename: review response.pdf [file pcbi.1007972.s011.pdf]

Response to reviewers

We thank the reviewers for their highly constructive comments, which we specifically respond to below (in blue). In brief, we have made the following major changes:

1) *Both reviewers raised several questions about figure 1, particularly figure 1B. We have substantially modified this figure (although the conclusions are unchanged), we hope this is now clearer.*
2) *Several questions were raised about why we chose to use an ABM, and why we chose the motility parameters that we did. To make this clearer, the previous figure 4 (using the Monte carlo simulations with dynamic conjugate formation) has been substantially modified and the position in the manuscript has been moved (now figure 2). We believe this ordering better motivates the ABM. Although we now do not fit the dynamic conjugation model directly to the ABM anymore, the broad conclusion of this figure is the same (the multiple-hitting model is not identifiable if the cellular interaction dynamics are unknown).*
3) *We have added procedures to test our multiple hitting hypothesis against the subpopulation hypothesis. Moreover, we also tested our model after incorporation of a minimal hitting time responding to suggestions from reviewer 2.*

Reviewer 1

Beck et al simulate CTL-mediated immune responses in different settings to better understand how CTL-mediated killing of target cells is regulated in vitro.
They use different simulation approaches to address the very important open question of how CTL-mediated killing of target cells can be better understood. This remains a very important question, given the high clinical importance of CTL-mediated immune responses against infectious diseases and cancer in the current age of immune-cell based therapeutic approaches.

We thank the reviewer for acknowledging the importance of the general set of questions our work aims to answer.

Regarding the Monte Carlo Simulations in Figure 1, it remains a little unclear how a small increase in simulated target cell numbers (plus 1) would change the outcome of the simulation in the way presented in Figure 1B (for the 100 hit case). Obviously, the “100 hit case” is a very extreme situation – but maybe this outcome of the simulation could be discussed and explained in more detail. Maybe a plotting of the effector-to-target cell ratio of these simulations would help to better understand this result?

Since both reviewers raised questions about Figure 1B, we decided to remove the extreme "100 hit" case as its physiological relevance is unclear, it could be confusing, and is not necessary for our conclusion. Moreover, based on the suggestion to show the effector-target ratio we have included two additional plots. In Figure 1G we now show a heatmap relating the initial number of targets to the number of targets killed. In our new Figure 2B we now also show the effector:target ratio within sample simulations.

Regarding the "over-dispersion" – maybe a short explanation of how such a conclusion was reached might be helpful (how was dispersion measured, and how can the cut-off that determines over-dispersion be justified).

We defined over-dispersion relative to the ratio of the variance and the mean for the Poisson distribution, which is expected to be one. This is now stated explicitly in the text corresponding to Figure 1.

Regarding the spatial model, it remains unclear how the "two CTL motility patterns" are used in the simulations: how are the two states set for individual cells? Does target cell contact decrease motility? Based on what experimental data are these motility features defined?

Our spatial simulations are intended to be realistic in the sense that they resemble the sort of datasets that are created from microscope imaging experiments, rather than being an attempt to offer mechanistic insight into the migratory behaviour of CTLs. The main role of these two different motility patterns was to achieve different killing kinetics, to ensure that our parameter estimation method would be able to cope with a range of killing kinetics. We have added a paragraph at the beginning of the results section on our CPM simulations to explain the aim of these simulations more explicitly.

Finally, to better understand how "cumulative contact time" in the simulations can be interpreted a short description of how the simulated CTL deliver the "lethal hits" in relation to contact duration would be helpful.

CTLs in all our simulation generate hits at a constant intrinsic rate, λ. This is the same for all simulations, although the rules for allocation of hits vary. These assumptions are now further clarified in our revised manuscript.

How is “hit delivery” defined in the spatial model? Could one contact event lead to multiple “hits”?

Multiple hits can indeed be delivered during the same contact. Note that in our revised manuscript we have also included an investigation of a minimal hitting time with our CPM, along with which we also explain our model of the hitting process more thoroughly.

Reviewer comments on Material and Methods section:

1.) Monte Carlo simulations:
How were these simulations performed? How was the data from Vasconcelos et al used exactly and where can the used data be found?

Besides explanation of the Monte Carlo simulations in the Methods, we have now also included more information on how these simulations were performed in the results section. With respect to the question how the data from Vasconcelos et al. (2015) were used , we only used general information that was reported in the paper. Note that we have also changed the way in which we used this general information and we explain this in detail in the revised version (in both the Results and the Methods).

How many simulations were performed?

We now include a summary table containing details about how many simulations and which types of simulation were performed.

2.) Spatial simulations
How was target cell motility defined? Why are the targets motile – how much does that affect the different conclusions (Maybe check results with non-motile and motile targets).

Targets were not modeled as actively motile, and their ‘passive’ motility is mostly caused by interaction with motile CTLs that drag them along. Simulation of active motility of targets would likely influence the observed distributions of interaction times, similar as CTL motility changes did. However, because we utilized the CPM simulations to test whether our methods can successfully estimate multi-hitting killing parameters from artificial data rather than to investigate potential influence of cellular motility on killing, we feel that adding the option of active target motility to our simulations does not contribute to the messages conveyed in this work. We have reworded our methods to make it clear that target cells were “moved” rather than “motile”.

Reviewer comments on Figures:

Figure 1:
Fig 1B: what does the Y-axis label really mean ("frequency")? Maybe this can be described in the figure legend.

We have avoided using this vague term in our revised manuscript, and strove to provide more explicit figure labels in Fig.1 and elsewhere.

Fig 1D: The Y axis is cut at 10 – is this really necessary?

This was not necessary and we apologise for our oversight.

Fig 1E: what does the Y-axis label mean – is it different to Fig 1B?

No, these quantities were in fact the same. As mentioned above, we took great care throughout our revised manuscript to provide clearer descriptions with our figures.

Figure 2:
Usually, target cell binding is expected to alter CTL migration – so how does target binding affect the CTL motility here?

The major focus of our study was to isolate and analyse the kinetics of CTL killing, without considering CTL motility directly. We have made this reasoning more explicit in the text where our spatial simulation is introduced (Figure 3). Moreover, we have incorporated additional discussion of CTL motility in Figure 3 of our revised submission.

How are the high vs low motility states "regulated" in these simulations?

High and low motility states were defined by the size of the ς_{Act} parameter, which is fixed ahead of the simulation thus there is no additional regulation of motility while the simulation is running. In our revised manuscript we explain this more clearly.

Reviewer 2

In this study, the authors use stochastic simulations to examine if heterogeneity in CTL killing dynamics can be explained by the requirement of target cells to receive multiple lytic hits. Using

explicit spatial simulations based on the CPM-framework, they further investigate how this hitting rate and the number of required hits per target cell can be inferred from imaging data. Based on their analyses they show that measuring the cumulative duration of CTL-target contacts would improve the accuracy of estimating CTL killing kinetics.
The topic is timely and relevant as understanding CTL efficacy is an important aspect for designing optimal therapeutic vaccination and treatment strategies, as e.g. against cancer. The study is well written and thoroughly structured. With this theoretical analysis the authors provide a reasonable explanation for previous observations from experimental studies and propose required measurements for further experiments. However, the study remains a bit limited by focusing on only one explanation for CTL killing dynamics.

We thank the reviewer for acknowledging the relevance and thoroughness of our work.

Major points:

(1.) The authors state that with the multiple-hitting-scenario, there is no need to invoke an unobserved subpopulation of high-rate killers (p. 22) as suggested by other studies. While this is true and nicely shown, I think the study would substantially benefit if the authors also show how the proposed information from imaging studies should differ in case of the latter scenario or others. It could be quite interesting to know on what type of measurements one has to focus in order to differ between different hypotheses, such as having cell populations that are heterogeneous (either in their killing efficacy (CTL) or susceptibility to killing (target cell)), having serial or multiple-killing, or assuming single- or multiple hit killing.

We agree with reviewer that the comparison to alternative models than multi-hit killing such as a model with subpopulations of low- and high-rate killers is useful. To this purpose, we also devised an approach to fit data to a model describing such subpopulations and to compare fits of the two models. We included this analysis in the supplement and briefly referred to it in the main text.

(2.) The ability to infer the hitting parameters in case of multiple-hitting CTL from CTL-target (cumulative) contact times in case of multiple-hitting is quite robust and interesting. However, measuring the cumulative contact times might be not sufficient in case that active conjugate formation would require the stopping of CTLs to allow them to deliver their lethal hits (Wiedemann et al., PNAS 2006), i.e., requiring a minimal contact time. This seems not to be directly implemented within the CPM as the hitting probability only considers the contact length for distributing the hit across multiple target cells.

We explicitly investigated the effect of a minimal hitting time and found that our estimates are expected to remain robust over a range of minimal hitting times (Fig. 6 and accompanying text). Moreover, we provide methods for checking the validity of the resulting estimates.

(3.) Another question concerns the parameterizations of the CPM. Do "high-" and "low-motility" CTL correspond in some way to the dynamics observed within the experiments or were they arbitrarily chosen?

These were chosen arbitrarily to represent contrasting but plausible scenarios, for which we investigated whether our method could successfully estimate multi-hit killing parameters. In the revised version we more explicitly discuss our rationale for the inclusion of these simulations.

(4.) Why is the average number of target cells killed after 12 hours for a density of 15 target cells higher when several hits are required compared to only one? This seems to be counterintuitive unless the hitting rates differ between the scenarios, maybe due to the adjustment done before and mentioned in the text? However, Figure 1E actually shows that for a given target cell concentration, the distribution gets shifted to the left the more hits are required.

The reviewer is correct that we adjusted both the number of hits required for killing and the hitting rate, i.e., we generally keep the ratio of the two constant. In the revised version we more explicitly explain this in the main text. Moreover, in our revised manuscript we used a consistent set of parameters throughout the manuscript, which should aid comparison between different settings.

The values of the rates used for simulation should be mentioned within the text or the figure legends. The corresponding Table (Table 1) seems to be empty.

Table 1 was merely meant to give an overview of the meaning of the utilised model parameters. In the revised version, for all figure panels we have clearly displayed the parameters inside the main text or legend.

Minor points:
- Figure 4A and Figure 5F are very difficult to read. Maybe there are better ways of representation, e.g. using different colors for each given (correct) parameter combination and similar colors with point sizes corresponding to frequencies for the estimates?

We have now focussed on only 3 parameter settings, which makes this figure easier to interpret. We also adopted the suggestion for the colour scheme (see Fig. 5E in revised version).

- Figure 1B: Does the red dotted line always refer to the 16 targets case for each row separately, i.e. for each number of hits (1,10,100)?

We changed Fig. 1 entirely to explain the key messages corresponding to this figure more clearly.

Have all data underlying the figures and results presented in the manuscript been provided? Large-scale datasets should be made available via a public repository as described in the *PLOS Computational Biology* data availability policy, and numerical data that underlies graphs or summary statistics should be provided in spreadsheet form as supporting information.
Reviewer #1: None
Reviewer #2: No: The parameters used for simulating the results shown in Figure 1 seem to be missing.

In the revised version, we have included parameter values for Fig. 1 in the legend as well as for all other figures in the methods, main text or figure legends. In addition, we have now provided a table providing an overview of the models and parameter settings used in the various figures. Finally, upon acceptance all data underlying the figures and all code will be uploaded to OSF.
